# Editorial: Eicosanoids in cancer, Volume II

**DOI:** 10.3389/fphar.2023.1224623

**Published:** 2023-06-14

**Authors:** Paola Patrignani, Emer M. Smyth, Emanuela Ricciotti

**Affiliations:** ^1^ Department of Neuroscience, Imaging, and Clinical Sciences and CAST, School of Medicine, “G. d’Annunzio” University, Chieti, Italy; ^2^ Department of Pathology and Cell Biology, Herbert Irving Comprehensive Cancer Center, Columbia University Medical Center, New York, NY, United States; ^3^ Department of Systems Pharmacology and Translational Therapeutics, Perelman School of Medicine, Institute for Translational Medicine and Therapeutics, University of Pennsylvania, Philadelphia, PA, United States

**Keywords:** eicosanoids, cancer, arachidonic acid, oxylipins, inflammation

Inflammation in the tumor0020microenvironment is now widely acknowledged as a hallmark of cancer (Hanahan, 2022). The tumor microenvironment is rich in both *de novo* synthetized and dietary fatty acids (FAs), including those derived from the oxidation of arachidonic acid and other polyunsaturated FAs known as eicosanoids.

Pro-inflammatory eicosanoids (including prostanoids and leukotrienes), which are produced by both tumor cells and stroma, can impact tumor progression through various mechanisms (Wang and Dubois, 2010). These biologically active lipids can influence tumor cell proliferation, apoptosis, migration, and invasion. They can also affect tumor neo-angiogenesis and alter tumor antigenicity, ultimately shaping the tumor microenvironment. Eicosanoids play a crucial role in the complex interplay between tumor cells, stroma, inflammatory cells, and platelets, opening new avenues of research for understanding the relationship between different pathophysiological mechanisms.

The eicosanoids in cancer, volume II, Research Topic combines: 1) a review presenting the current understanding of the role of FAs in colorectal cancer progression (Hoxha and Zappacosta); 2) a meta-analysis of randomized clinical trials on the efficacy of omega-3 polyunsaturated FAs in colorectal cancer (Liu et al.); 3) two articles presenting cyclooxygenase (COX)-2 dependent and independent approaches to improve the sensitivity to chemotherapy (Lin et al.) or to reduce inflammation and oxidative stress (Apweiler et al.).

A comprehensive review by Hoxha and Zappacosta focuses on the involvement of FAs and their metabolites in colorectal cancer. Data presented in the review indicates that FAs may contribute to the development and progression of colorectal cancer through several mechanisms. The authors, therefore, suggest that enzymes involved in FA metabolism could represent novel therapeutic targets for adjuvant therapy in cancer patients.

A meta-analysis by Liu et al. systematically evaluated the efficacy of omega-3 polyunsaturated FAs on several clinical parameters associated with colorectal cancer progression. The meta-analysis reveals that omega-3 supplementation reduces the level of some inflammatory cytokines and shortens the length of hospitalization but does not affect the outcome of postoperative colorectal cancer patients.

COX-2 is often implicated in the chemoresistance of certain malignant tumors, and its inhibition with nonsteroidal anti-inflammatory drugs (NSAIDs) may enhance the sensitivity of tumors to anticancer drugs (Bell et al., 2022). Lin et al. report a novel molecular mechanism of COX-2 upregulation in chemoresistant non-small cell lung cancer (NSCLC) cells. The authors show that a reactive oxygen species-ERK1/2-NF-κB signaling axis and prostaglandin (PG)E_2_-PGE_2_ receptors-ERK1/2 positive feedback loop are involved in the COX-2 upregulation by cisplatin, which induces multidrug resistance of NSCLC cells through upregulation of BCL2 expression and the subsequent attenuation of cell apoptosis. COX-2 inhibition with the NSAID celecoxib reverses the chemoresistance of NSCLC cells *in vitro* and *in vivo*.

Apweiler et al. report that another NSAID, acetaminophen, and its metabolite, AM404, can reduce interleukin-1β-induced PGE_2_ generation through a COX-independent pathway that appears linked to reductions in oxidative stress in SK-N-SH neuroblastoma cells.

Our knowledge of the relationship between FAs and cancer is incomplete. It is necessary to research further the various types of FAs and their metabolites to understand their clinical significance in cancer diagnosis and treatment. While we have more information on the impact of COX-2 and its metabolites on cancer initiation, progression, metastasis, and resistance, the use of NSAIDs combined with chemotherapy or immunotherapy has not been well established yet.
